# Therapeutic drug monitoring of lacosamide among children: is it helpful?

**DOI:** 10.3389/fphar.2023.1164902

**Published:** 2023-07-06

**Authors:** Elkana Kohn, Mirit Lezinger, Sharon Daniel, Majdi Masarwi, Nurit Brandriss, Adina Bar-Chaim, Matitiahu Berkovitch, Eli Heyman, Rinat Komargodski

**Affiliations:** ^1^ Clinical Pharmacology and Toxicology Unit, Shamir Medical Center (Assaf Harofeh), Zerifin, Israel; ^2^ Pediatric Neurology Department, Shamir Medical Center (Assaf Harofeh), Zerifin, Israel; ^3^ Department of Public Health and Pediatrics, Ben-Gurion University of the Negev and Clalit Health Services, Beer-Sheva, Israel; ^4^ Pharmacy Services, Shamir Medical Center (Assaf Harofeh), Zerifin, Israel; ^5^ Laboratories Department, Biochemistry Lab, Shamir Medical Center (Assaf Harofeh), Zerifin, Israel; ^6^ Clinical Pharmacology and Toxicology Unit, Shamir Medical Center (Assaf Harofeh), Zerifin and Sackler Faculty of Medicine, Tel-Aviv University, Tel-Aviv, Israel; ^7^ Pharmacy Services, Shamir Medical Center (Assaf Harofeh), School of Pharmacy, Faculty of Medicine, The Hebrew University of Jerusalem, Jerusalem, Israel

**Keywords:** therapeutic drug monitoring (TDM), plasma concentration, lacosamide (LCM), pediatric population, seizures, epilepsy

## Abstract

**Objective:** This study aimed to investigate the efficacy and tolerability of Lacosamide (LCM) in a pediatric population with epilepsy using LCM serum concentration and its correlation to the age of the participants and the dosage of the drug.

**Methods:** Demographic and clinical data were collected from the medical records of children with epilepsy treated with LCM at Shamir Medical Center between February 2019 to September 2021, in whom medication blood levels were measured. Trough serum LCM concentration was measured in the biochemical laboratory using High-Performance Liquid Chromatography (HPLC) and correlated with the administered weight-based medication dosing and clinical report.

**Results:** Forty-two children aged 10.43 ± 5.13 years (range: 1–18) were included in the study. The average daily dose of LCM was 306.62 ± 133.20 mg (range: 100–600). The average number of seizures per day was 3.53 ± 7.25 compared to 0.87 ± 1.40 before and after LCM treatment, respectively. The mean LCM serum concentration was 6.74 ± 3.27 mg/L. No statistically significant association was found between LCM serum levels and the clinical response (*p* = 0.58), as well as the correlation between LCM dosage and the change in seizure rate (*p* = 0.30). Our study did not find a correlation between LCM serum concentration and LCM dosage and the gender of the participants: males (n = 17) females (n = 23) (*p* = 0.31 and *p* = 0.94, respectively). A positive trend was found between age and LCM serum concentrations (r = 0.26, *p* = 0.09).

**Conclusion:** Based on the data that has been obtained from our study, it appears that therapeutic drug monitoring for LCM may not be necessary. Nonetheless, further research in this area is needed in the light of the relatively small sample size of the study.

## Introduction

Lacosamide (LCM) is a third-generation anti-seizure medication (ASM) approved in 2008 by the FDA as an add-on therapy for treating focal-onset seizures in people with epilepsy who are 17 years old and older. In 2020, the FDA extended the indication to include LCM as adjunctive therapy in patients 4 years of age and older to treat primary generalized tonic-clonic seizures (Vimpat, 2022). In Israel, LCM has been included in healthcare services since 2014.

The mechanism of action of LCM is not fully elucidated. *In vitro* electrophysiological studies have shown that LCM selectively enhances the slow inactivation of voltage-gated sodium channels, resulting in the stabilization of hyperexcitable neuronal membranes (FDA, 2022).

LCM has linear pharmacokinetics. It is rapidly absorbed after oral administration, and has a bioavailability of approximately 100%. The plasma concentration reaches its maximum level 1–2 h after dosing, and it has a volume of distribution of around 0.6–0.7 L/kg, with less than 14% bound to plasma proteins. LCM undergoes hepatic metabolism through demethylation and is renally excreted at a rate of up to 95%. The half-life of LCM in children over 4 years old depends on weight: 11 kg–7.4 h, 28.9 kg–10.6 h, 70 kg–14.8 h, respectively (Patsalos et al., 2018), (Doty et al., 2007).

Currently there is no recommendation for LCM therapeutic drug monitoring (TDM) (FDA, 2023), (Schultz and Mahmoud, 2020). However, TDM can help determine an individual’s optimal serum/plasma concentration range and identify the serum concentration levels at which seizures are controlled or ASM-specific adverse effects occur. This study aimed to evaluate the efficacy and tolerability of LCM in a pediatric population with epilepsy in relation to serum concentration, dose, adverse effects, gender, and age.

## Patients and methods

The Ethics Committee approved the study at Shamir Medical Center. The LCM serum concentrations were measured in the hospital’s biochemistry lab using a commercial kit (Chromysystems) and high-performance liquid chromatography with photodiode array ultraviolet detection (Variant Prostar). LCM reference levels were 1–10 mg/L (Hiemke et al., 2011). Patients were seen by a neurologist every 3 months as part of routine care, and blood for LCM serum levels was taken a month after treatment initiation, a change in dosage, or after IV administration of a loading dose. If the patient did not show clinical improvement, the dose was increased or discontinued. The last available serum concentration of each patient was included in the study, as it represents steady state. The laboratory database of Shamir Medical Center was searched for LCM blood level samples from children with epilepsy treated with the medication as monotherapy or polytherapy from February 2019 to September 2021. Medical records were obtained from the pediatric neurology department. The criteria for inclusion in the study were patients aged 0–18 years with epilepsy who were treated with LCM for any type of seizures and had recorded LCM blood levels during the study period. Patients with cancer, neurodegenerative diseases, major deformations in the central nervous system, pseudo-seizures and metabolic diseases were excluded from the study. Patients whose LCM blood levels were taken when receiving loading dose were not included in the analysis. Data, including LCM blood levels, dose per weight, seizure frequency before and after treatment, concomitant ASMs, adverse effects, and demographic information such as age, gender, weight, height, birth complications, usage of illicit drugs, age of onset of epilepsy, type of epilepsy and its origin, genetic disorders, and family history of chronic diseases including epilepsy, were collected from the patient’s medical records. Patients’ parents were instructed to follow the frequency of seizures. Parents’ reports were documented in medical records before and during LCM treatment when attending regular clinic appointments. Patients who were considered seizure-free were those for whom the parents reported no occurrences of seizures whatsoever in addition to the neurologist evaluation and the EEG results (Kwan et al., 2010).

## Statistical analysis

All statistical analysis was conducted using R Statistical Software, version 3.5.2 (Foundation for Statistical Computing, Vienna, Austria). The data was described using means, standard deviations, minimum, maximum, rates, and percentages for quantitative and categorical variables, respectively. The χ2 and Fisher exact tests were utilized to compare between groups for categorical outcomes and the independent samples t-test for quantitative outcomes. The Pearson and Spearman correlations were used to correlate between outcomes. Finally, the paired t-test was used to evaluate the difference in seizure frequency before and after starting LCM treatment.

## Results

**FIGURE 1:**
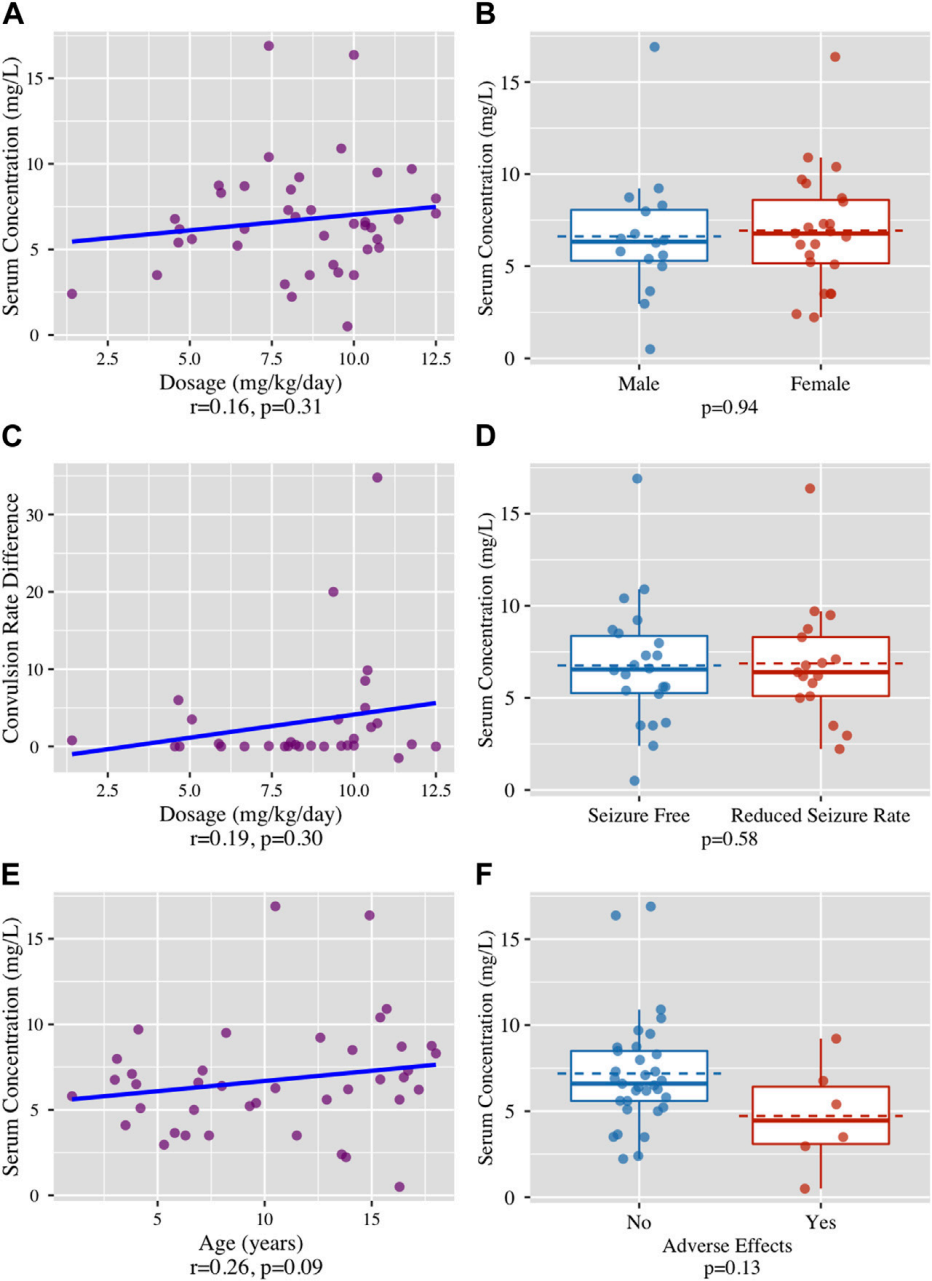
**(A)** No correlation found between LCM dosage (mg/kg/day) and LCM serum concentrations (mg/L), r = 0.16, *p* = 0.31. **(B)** No correlation found between the patient’s gender and LCM serum concentrations (mg/L), (*p* = 0.94). **(C)** No correlation found between The LCM dosage (mg/kg/day) and the clinical effect in terms of the difference in daily seizure rate, (r = 0.19, *p* = 0.3). **(D)** No correlation found between clinical response, defined as absolute seizure free or seizures rate reduction and LCM serum concentrations (mg/L), (*p* = 0.58) **(E)** No correlation found between participants’ age (years) and LCM serum concentrations (mg/L), (r = 0.26, *p* = 0.09). **(F)** No correlation found between incidence of adverse effects and LCM serum concentrations (mg/L), (*p* = 0.13).

Forty-two patients were screened. Two patients were excluded as the LCM blood levels were not taken at trough. Of the included patients, 43% (n = 17) were boys and 57% (n = 23) were girls. The average age of the patients was 10.4 ± 5.1 years (range: 1–18). Twenty eight patients (61%) had a focal onset epilepsy (24 patients with Drug-resistant refractory focal epilepsy, 2 with Tuberous sclerosis, 2 with Electrical Status Epilepticus in Sleep (ESES) and one with Drug-resistant temporal lobe epilepsy). Seven patients (15%) had a Generalized onset epilepsy (4 patients with Generalized convulsive epilepsy and 3 with Absence). Eleven patients (24%) had a combined generalized and focal onset epilepsy. The average age at diagnosis was 6.41 ± 4.81 years (0–16). Thirty-nine patients were diagnosed with refractory epilepsy failing to achieve sustained seizure freedom on at least two tolerated, appropriately chosen and used ASMs were taking an average of 3.8 ± 2.1 (range 0–10) ASMs before starting LCM therapy. At the time of LCM treatment, the average number of concurrent ASMs was 1.1 ± 0.96 (range 0–4). One patient with a focal onset epilepsy was treated with LCM as a first-line monotherapy. Before the treatment, the average frequency of seizures was 3.5 ± 7.2 per day (range: 1–35). The total daily dose of LCM was 306.62 ± 133.20 mg (range: 100–600). The average blood level of LCM was 6.74 ± 3.27 mg/L (range: 0.7–16.9). Four (10%) had serum concentration above upper reference value of 10 mg/L. Seizure frequency decreased significantly following the initiation of LCM treatment (*p* = 0.02). While 65% of the patients (n = 26) were seizure-free, reported an average period of 229 ± 203 days (range 33–943), 35% of the patients (n = 14) had reduction in convulsions rate, suffering from an average of 0.87 ± 1.4 (range: 1–3.5) seizures per day (Tables 1, 2 and Figure 1D). LCM serum concentrations were not significantly correlated with LCM weight-based dosage (r = 0.16, *p* = 0.31), gender (*p* = 0.94) and the convulsion rate difference (r = 0.19, *p* = 0.3) (Figures 1A–C). No statistically significant association was found between LCM serum levels and the clinical response (*p* = 0.58) (Figure 1D). A positive trend, but not statistically significant, was found between age and LCM serum concentrations (r = 0.26, *p* = 0.09) (Figure 1E). Adverse effects were reported in 7 (18%) patients, and included dizziness (n = 2), drowsiness (n = 1), other (n = 4). Adverse effects were not correlated with LCM serum levels (*p* = 0.13) (Figure F). We calculated the ratio between serum concentration and weight adjusted daily dose (C/D ratio). No significant differences in C/D ratios were found between the “seizure free” group and the “less convulsions” group (C/D ratio means of 0.93 and 0.80 respectively, *p* = 0.40), between male and female (means of 0.82 and 0.92 respectively, *p* = 0.52) and between children with and without ADRs (means of 0.92 and 0.60 respectively, *p* = 0.15). The correlation between C/D ratio and convulsion rate difference was negative but not statistically significant (r = −0.16, *p* = 0.38). A positive and statistically significant correlation was found between C/D ratio and age (r = 0.49, *p* < 0.001).

**TABLE 1:** Demographic and baseline characteristics of the participants (*n* = 40). Weight, height and BMI are presented for each age group. Values are presented as mean ± SD (range) or n (%).

Demographic characteristics	
Female	23 (57%)
Male	17 (43%)
**Age, years**	10.43 ± 5.13 (1–18)
1–5 years	n = 13 (32%)
6–11 years	n = 9 (23%)
12–18 years	n = 18 (45%)
**Weight, Kg**	
1–5 years	18.66 ± 4.91 (11–29)
6–11 years	35.33 ± 11.5 (20–54)
12–18 years	60.75 ± 19.49 (28–96)
**Height, m**	
1–5 years	1.05 ± 0.13 (0.76–1.29)
6–11 years	1.31 ± 0.11 (1.1–1.48)
12–18 years	1.59 ± 0.10 (1.4–1.76)
**BMI, kg/m^2^**	
1–5 years	16.44 ± 1.45 (14.4–19.09)
6–11 years	21.05 ± 8.84 (12.8–42.97)
12–18 years	24.14 ± 7.99 (13.7–44.42)

**TABLE 2:** Medical characteristics of the participants (*n* = 40). Values are presented as mean ± SD (range) or n (%).

Medical characteristics	
**Epilepsy Type:** Focal Onset	28 (61%)
**Epilepsy Type:** Generalized Onset	7 (15%)
**Epilepsy Type:** Combined Generalized and Focal Onset	11 (24%)
**Age at diagnosis, years**	6.41 ± 4.81 (0–16)
**Number of ASMs before LCM therapy**	3.8 ± 2.1 (0–10)
**Number of Concomitant ASMs**	1.1 ± 0.96 (0–4)
**Number of Patients taking LCM as a monotherapy**	10 (25%)
**LCM dosage, mg/kg/day**	8.40 ± 2.51 (1.40–12.5)
**Total daily dose, mg**	306.62 ± 133.20 (100–600)
**LCM serum concentration, mg/L**	6.74 ± 3.27 (0.7–16.9)
**Concentration to dosage (C/D ratio)**	0.88 ± 0.47 (0.01–2.28)
**Seizure per day before LCM**	3.53 ± 7.25 (0.002–35)
**Seizure per day after LCM**	0.87 ± 1.40 (0.002–3.5)
**Adverse Effects**	7 (18%)

## Discussion

No correlation was found between LCM serum levels and clinical efficacy and tolerability. Moreover, our study found no correlation between administered dosage and serum drug level. Only few studies have evaluated the correlation between LCM dose and serum concentrations (Ben-Menachem et al., 2007; Greenaway et al., 2011; Svendsen et al., 2017; Reimers et al., 2018; Schultz and Mahmoud, 2020). Those studies were primarily conducted on adults. According to *Greenaway’s* prospective observational study that was conducted on 98 patients (average age: 43 ± 12 years) with a mean LCM daily dose of 3.3 ± 1.6 mg/kg, LCM dose was linearly related to total and free LCM serum concentrations (*r* ^2^ = 0.825, 0.815, respectively) (Greenaway et al., 2011). A weak positive correlation was also found in *Svendsen et al.* study (age 4–86 years, n = 344) that investigated the correlation between dose and serum concentration (*r* ^2^ = 0.1779, *p* < 0.05). The mean LCM serum concentration was 4.7 ± 2.5 mg/L (range: 1–17.3 mg/L) (Svendsen et al., 2017). However, these results were not analyzed specifically for children. A weak relationship (r = 0.238) was found between the LCM daily dose and LCM plasma concentration in a recent study that was performed on 500 Chinese pediatric patients (Zhao et al., 2023). The differences between our findings and those of previous studies could be due to the different physiological characteristics of children and adults. For example, the total body water is higher in children compared to adults, which alters the volume of distribution, changes in stomach acidity that might affect the absorption of medications, and changes in drug metabolism throughout pediatric age groups (Accesspharmacy, 2022). All these factors might affect the correlation between LCM dose and blood concentrations. There is limited data available on the correlation between LCM plasma concentration and clinical efficacy. A study by *Chung et al.*, analyzed data from three clinical trials on adult patients with partial-onset seizures (n = 1,294) and found that the Emax model, which measures the maximum number of seizures, was the most appropriate for describing the relationship between AUC and seizure frequency change. No therapeutic serum concentration range has been established for LCM (Chung et al., 2010). Although a linear correlation between LCM plasma levels and the clinical response was not found in our study, other studies reported a correlation between LCM dosage and therapeutic efficacy in the pediatric population (Casas-Fernández et al., 2012; Verrotti et al., 2013; Sanmartí-Vilaplana and Díaz-Gómez, 2018; Hmaimess et al., 2020). The therapeutic dose used in our study was 1.4–12.5 mg/kg/day, which falls within the effective dose range of 1.6–20 mg/kg/day as reported in literature (Zhao et al., 2021). The maximum daily seizure rate before treatment with LCM was significantly higher than the daily rate after treatment, this brings out a possibility of outliers which are causing the significant difference before and after treatment. We performed a sensitivity analysis excluding every case with a daily seizure rate of 5 and more before treatment and achieved similar results (*p* = 0.003). Data on the LCM reference range is scarce and is not well established. The recommended reference ranges have been reported as varying from 2.2 to 20 mg/L (Schultz and Mahmoud, 2020). The LCM plasma concentrations in our study were found to be in the range of 0.7–16.9 mg/L with a mean of 6.74 ± 3.27 mg/L. In Norway and Denmark, the LCM serum concentration reference range is 3–10 mg/L (Reimers et al., 2018). In *Zhao et.al* study, LCM plasma concentrations of patients ranged from 1.5 to 19.7 mg/L, with a mean of 6.9 ± 3.2 mg/L (Zhao et al., 2023).

Our study found no significant correlation between LCM serum concentration and gender. However, a study by *Markoula S. et al.* on an adult population (women: n = 68, men: n = 61) aged 19–66 years and taking a median LCM dose of 300 mg found that women had a higher mean LCM concentration (9.3 ± 5.9 mg/L) compared to men (6.7 ± 3.2 mg/L), with a statistically significant difference (*p* = 0.001) (Markoula et al., 2014). The current literature on the pharmacokinetics of LCM in pediatric population is limited. Our study found a positive trend between age and LCM serum concentrations. This result is in accordance with previous findings (Larsen Burns et al., 2019), (May et al., 2018). According to *L. Burns’* study based on databases at two national epilepsy centers in Norway and Denmark, children aged between 13 and 17 years (n = 44) had significantly higher concentration–dose ratios than children aged between 6 and 12 years (n = 29) and children aged<6 years (n = 3) (Larsen Burns et al., 2019). It is plausible that it reflects an increased clearance of LCM, considering the age-related physiological changes through childhood (Batchelor and Marriott, 2015), (Johannessen Landmark et al., 2012). *Zaho et al.* also presented similar findings, indicating that the age group of 6–14 years had significantly higher LCM levels (*p* < 0.001) (Zhao et al., 2023). Our study found that 18% of patients experienced side effects while taking LCM. This is in line with previous studies that reported incidences ranging from 18% to 59%. The most common side effects were dizziness, sedation, gastrointestinal upset, mood changes, and behavioral changes (Hmaimess et al., 2020), (Verrotti et al., 2013), (Farkas et al., 2019), (Ortiz de la Rosa et al., 2018) and all side effects were tolerable, similar to our findings. Our study did not find a correlation between adverse effects and LCM serum levels. This result is controversial in the literature. According to *Buck et al.*, LCM’s adverse effects in children have been classically associated with higher dosages and speed of titration (Buck and Goodkin, 2012). However, most studies, although conducted on an adult population, did not find a significant difference in plasma concentration between those who experienced adverse effects and those who did not (Casas-Fernández et al., 2012), (Hillenbrand et al., 2011), (Mcginnis and Kessler, 2016).

## Study limitations

The study was limited to children who received LCM for at least 1 month at a therapeutic dose and showed initial improvement in seizure frequency. Children who experienced an increase or no decrease in seizure frequency during treatment with LCM were not included in the study.

Our study did not examine the effect of co-administered ASMs on LCM serum levels and efficacy. Studies suggest that the efficacy and tolerability of LCM may decrease if added to a regimen containing sodium channel blockers (Farkas et al., 2019), (Yamamoto et al., 2020). *Winkler et al*. study examined LCM use in pediatrics found that LCM clearance increased by about 35% when administered with a concomitant enzyme-inducer AEDs (Winkler et al., 2019). *May et al.* observed a reduction of 46% in the AUC of LCM when children were given enzyme-inducer AEDs in combination with LCM compared to monotherapy (May et al., 2018).

## Conclusion

Based on the current data, it appears that measuring serum concentrations of LCM in pediatric patients undergoing treatment might not be necessary. However, monitoring LCM levels can still play a role in the management of patients, especially those with refractory epilepsy to assess compliance, determine a therapeutic baseline in patients who have well-controlled seizures, or identify severe intoxication. Further research in this area is needed among pediatric patients to confirm these findings.

## Data Availability

The raw data supporting the conclusion of this article will be made available by the authors, without undue reservation.
